# (3,6-Dibromo-*o*-phenyl­ene)dimethanol

**DOI:** 10.1107/S1600536811001206

**Published:** 2011-01-15

**Authors:** Qi Li, Wei-jian Xue

**Affiliations:** a Key Laboratory of Pesticides and Chemical Biology of the Ministry of Education, College of Chemistry, Central China Normal University, Wuhan 430079, People’s Republic of China

## Abstract

The title compound, C_8_H_8_Br_2_O_2_, was synthesized from the hydrolysis of 1,4-dibromo-2,3-bis­(bromo­meth­yl)benzene. One intra­molecular O—H⋯O  and two intra­molecular C—H⋯Br inter­actions occur. In the crystal, mol­ecules are linked into a chain running parallel to [010]. Adjacent chains are linked into a two-dimensional layer by a combination of inter­molecular O—H⋯O hydrogen bonds and C—H⋯π inter­actions.

## Related literature

For the preparation of the title compound, see: Abad (2005 ▶); Lai & Yap, (1993 ▶).
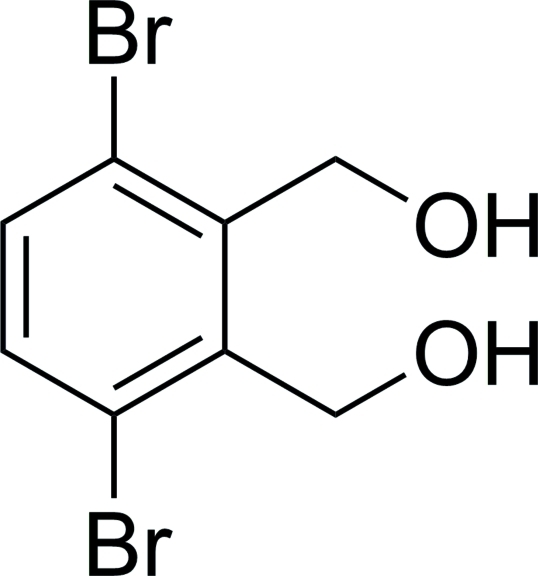


## Experimental

### 

#### Crystal data

C_8_H_8_Br_2_O_2_
*M* *_r_* = 295.96
Orthorhombic, 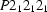

*a* = 4.482 (2) Å
*b* = 9.123 (5) Å
*c* = 22.819 (11) Å
*V* = 933.0 (8) Å^3^
*Z* = 4
Mo *K*α radiation
μ = 8.64 mm^−1^
*T* = 298 K
0.20 × 0.10 × 0.10 mm

#### Data collection

Bruker SMART APEX diffractometer
Absorption correction: multi-scan (*SADABS*; Sheldrick, 2008 ▶) *T*_min_ = 0.277, *T*_max_ = 0.479
11471 measured reflections
2300 independent reflections
1795 reflections with *I* > 2σ(*I*)
*R*_int_ = 0.040

#### Refinement

*R*[*F* ^2^ > 2σ(*F* ^2^)] = 0.043
*wR*(*F* ^2^) = 0.111
*S* = 1.05
2300 reflections
115 parameters
2 restraints
H atoms treated by a mixture of independent and constrained refinement
Δρ_max_ = 1.17 e Å^−3^
Δρ_min_ = −0.60 e Å^−3^
Absolute structure: Flack (1983 ▶), 918 Friedel pairs
Flack parameter: 0.03 (2)

### 

Data collection: *SMART* (Bruker, 1997 ▶); cell refinement: *SAINT* (Bruker, 1999 ▶); data reduction: *SAINT*; program(s) used to solve structure: *SHELXS97* (Sheldrick, 2008 ▶); program(s) used to refine structure: *SHELXL97* (Sheldrick, 2008 ▶); molecular graphics: *SHELXTL* (Sheldrick, 2008 ▶); software used to prepare material for publication: *SHELXL97*.

## Supplementary Material

Crystal structure: contains datablocks I, global. DOI: 10.1107/S1600536811001206/om2391sup1.cif

Structure factors: contains datablocks I. DOI: 10.1107/S1600536811001206/om2391Isup2.hkl

Additional supplementary materials:  crystallographic information; 3D view; checkCIF report

## Figures and Tables

**Table 1:** Hydrogen-bond geometry (Å, °) *Cg*1 is the centroid of the C1–C6 ring.

*D*—H⋯*A*	*D*—H	H⋯*A*	*D*⋯*A*	*D*—H⋯*A*
C8—H8*A*⋯Br2	0.97	2.61	3.192 (6)	119
C7—H7*B*⋯Br1	0.97	2.66	3.169 (5)	114
O2—H2⋯O1^i^	0.82 (6)	1.92 (3)	2.703 (6)	160 (7)
O1—H1⋯O2	0.81 (6)	1.95 (4)	2.703 (6)	152 (7)
C8—H8*B*⋯*Cg*1^ii^	0.97	3.01	3.63(5)	123

Symmetry codes: (i) 
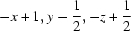
; (ii) 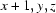.

**Fig. 1.**
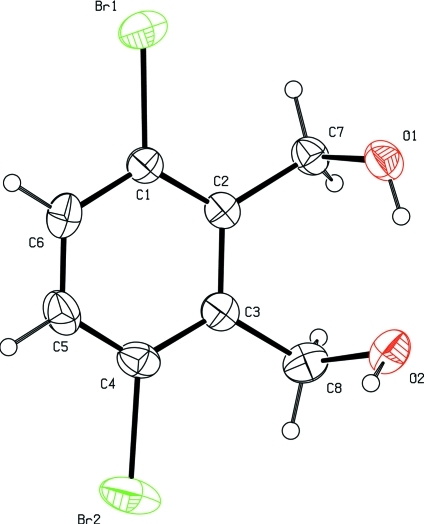
A view of the title compound, showing the atom labelling scheme, with displacement ellipsoids drawn at the 30% probability level. H atoms are omitted for clarity.

## supplementary crystallographic information

### Comment

Benzyl alcohol derivatives are important compounds as preservatives, dyes, fibers, and nylons. In our work, the hydrolysis of 1,4-dibromo-2,3-bis(bromomethyl)benzene gave rise to the dimethanol product reported here. The molecules of (3,6-dibromo-1,2-phenylene)dimethanol (Figure 1) are linked into a one-dimensional chain running parallel to the [010] direction by intra- and intermolecular O-H···O hydrogen bond interactions. The adjacent chains are linked into a two-dimensional layer by the combination of the O2—H2···O1(1 - *x*, *y* - 1/2, 1/2 - *z*) (Table 1) hydrogen bonds and C—H···π interaction (H8B (1+x,y,z)···*Cg*1 = 3.01 (1) Å, C8—H8B···*Cg*1 = 122.9°, *Cg*1 is the centroid defined by atoms C1—C6.)

### Experimental

The title compound was synthesized according to the reported literature (Abad *et al.*, 2005 and Lai *et al.*, 1993). The reaction of 1,4-dibromo-2,3-dimethylbenzene with N-bromosuccinimide in carbon tetrachloride in the presence of benzoyl peroxide under illumination and reflux gave 1,4-dibromo-2,3-bis(bromomethyl)benzene. Then 1,4-dibromo-2,3-bis(bromomethyl)benzene with NaOH in water at room temperature gave rise to (3,6-dibromo-1,2-phenylene)dimethanol . Crystals suitable for X-ray diffraction were grown by slow evaporation of a chloroform-methanol (5:1) solution of the title compound at room temperature.

### Refinement

All carbon H atoms were placed at their idealized positions with C-H = 0.93 Å (aromatic), 0.97 Å (methylene)) and *U*_iso_(H) =1.2 *U*_eq_(C) (aromatic and methylene). Hydrogen atoms bonded oxygen atoms were located from the Fourier difference maps and refined with the restraints of O—H=0.82 (1) Å and *U*_iso_(H) =1.5 *U*_eq_(O).

### Crystal data

**Table:**

C_8_H_8_Br_2_O_2_	*F*(000) = 568
*M**_r_* = 295.96	*D*_x_ = 2.107 Mg m^−^^3^
Orthorhombic, *P*2_1_2_1_2_1_	Mo *K*α radiation, λ = 0.71073 Å
Hall symbol: P 2ac 2ab	Cell parameters from 3167 reflections
*a* = 4.482 (2) Å	θ = 2.4–24.1°
*b* = 9.123 (5) Å	µ = 8.64 mm^−^^1^
*c* = 22.819 (11) Å	*T* = 298 K
*V* = 933.0 (8) Å^3^	Needle, colorless
*Z* = 4	0.20 × 0.10 × 0.10 mm

### Data collection

**Table:**

Bruker SMART APEX diffractometer	2300 independent reflections
Radiation source: fine-focus sealed tube	1795 reflections with *I* > 2σ(*I*)
graphite	*R*_int_ = 0.040
φ and ω scans	θ_max_ = 28.3°, θ_min_ = 2.4°
Absorption correction: multi-scan (*SADABS*; Sheldrick, 2008)	*h* = −5→5
*T*_min_ = 0.277, *T*_max_ = 0.479	*k* = −12→12
11471 measured reflections	*l* = −30→30

### Refinement

**Table:**

Refinement on *F*^2^	Secondary atom site location: difference Fourier map
Least-squares matrix: full	Hydrogen site location: inferred from neighbouring sites
*R*[*F*^2^ > 2σ(*F*^2^)] = 0.043	H atoms treated by a mixture of independent and constrained refinement
*wR*(*F*^2^) = 0.111	*w* = 1/[σ^2^(*F*_o_^2^) + (0.0525*P*)^2^ + 0.6273*P*] where *P* = (*F*_o_^2^ + 2*F*_c_^2^)/3
*S* = 1.05	(Δ/σ)_max_ = 0.004
2300 reflections	Δρ_max_ = 1.17 e Å^−^^3^
115 parameters	Δρ_min_ = −0.60 e Å^−^^3^
2 restraints	Absolute structure: Flack (1983), 918 Friedel pairs
Primary atom site location: structure-invariant direct methods	Flack parameter: 0.03 (2)

### Special details

**Table:**

Geometry. All e.s.d.'s (except the e.s.d. in the dihedral angle between two l.s. planes) are estimated using the full covariance matrix. The cell e.s.d.'s are taken into account individually in the estimation of e.s.d.'s in distances, angles and torsion angles; correlations between e.s.d.'s in cell parameters are only used when they are defined by crystal symmetry. An approximate (isotropic) treatment of cell e.s.d.'s is used for estimating e.s.d.'s involving l.s. planes.
Refinement. Refinement of *F*^2^ against ALL reflections. The weighted *R*-factor *wR* and goodness of fit *S* are based on *F*^2^, conventional *R*-factors *R* are based on *F*, with *F* set to zero for negative *F*^2^. The threshold expression of *F*^2^ > σ(*F*^2^) is used only for calculating *R*-factors(gt) *etc*. and is not relevant to the choice of reflections for refinement. *R*-factors based on *F*^2^ are statistically about twice as large as those based on *F*, and *R*- factors based on ALL data will be even larger.

### Fractional atomic coordinates and isotropic or equivalent isotropic displacement parameters (Å^2^)

**Table:**

	*x*	*y*	*z*	*U*_iso_*/*U*_eq_	
Br1	0.30061 (15)	0.81127 (6)	0.06905 (3)	0.0657 (2)	
Br2	0.8120 (2)	0.15549 (7)	0.12029 (4)	0.0930 (3)	
C1	0.4665 (11)	0.6246 (5)	0.0868 (2)	0.0422 (11)	
C2	0.6474 (10)	0.6045 (5)	0.13420 (19)	0.0380 (10)	
C3	0.7599 (11)	0.4622 (5)	0.14561 (19)	0.0443 (11)	
C4	0.6766 (13)	0.3496 (5)	0.1078 (2)	0.0518 (12)	
C5	0.4952 (14)	0.3730 (7)	0.0605 (2)	0.0593 (14)	
H5	0.4439	0.2955	0.0360	0.071*	
C6	0.3902 (13)	0.5100 (7)	0.0495 (2)	0.0532 (13)	
H6	0.2683	0.5270	0.0172	0.064*	
C7	0.7251 (12)	0.7281 (5)	0.17504 (19)	0.0466 (11)	
H7A	0.9324	0.7197	0.1868	0.056*	
H7B	0.7003	0.8209	0.1549	0.056*	
C8	0.9546 (13)	0.4374 (7)	0.1983 (2)	0.0566 (13)	
H8A	1.0251	0.3369	0.1982	0.068*	
H8B	1.1273	0.5013	0.1960	0.068*	
O1	0.5374 (9)	0.7250 (4)	0.22612 (15)	0.0555 (9)	
H1	0.582 (16)	0.651 (5)	0.244 (3)	0.083*	
O2	0.7969 (9)	0.4656 (4)	0.25204 (16)	0.0610 (9)	
H2	0.680 (14)	0.397 (6)	0.250 (3)	0.091*	

### Atomic displacement parameters (Å^2^)

**Table:**

	*U*^11^	*U*^22^	*U*^33^	*U*^12^	*U*^13^	*U*^23^
Br1	0.0672 (3)	0.0603 (3)	0.0697 (3)	0.0117 (3)	0.0053 (3)	0.0190 (3)
Br2	0.1119 (6)	0.0401 (3)	0.1269 (6)	0.0091 (4)	0.0183 (5)	0.0023 (3)
C1	0.042 (3)	0.042 (3)	0.043 (2)	−0.004 (2)	0.006 (2)	0.0020 (19)
C2	0.031 (2)	0.040 (2)	0.042 (2)	−0.0079 (18)	0.0103 (19)	−0.0006 (18)
C3	0.041 (3)	0.046 (2)	0.046 (2)	−0.006 (2)	0.013 (2)	0.0019 (19)
C4	0.054 (3)	0.039 (2)	0.062 (3)	−0.005 (2)	0.010 (3)	0.002 (2)
C5	0.070 (4)	0.053 (3)	0.056 (3)	−0.014 (3)	−0.001 (3)	−0.015 (2)
C6	0.052 (3)	0.067 (3)	0.041 (2)	−0.010 (2)	−0.004 (2)	0.001 (2)
C7	0.047 (3)	0.043 (2)	0.050 (2)	−0.006 (2)	0.004 (2)	−0.0046 (19)
C8	0.045 (3)	0.060 (3)	0.065 (3)	0.002 (3)	0.000 (3)	0.009 (3)
O1	0.066 (2)	0.049 (2)	0.052 (2)	−0.0007 (19)	0.0113 (19)	−0.0068 (16)
O2	0.062 (2)	0.069 (2)	0.0515 (18)	−0.007 (2)	−0.004 (2)	0.0116 (18)

### Geometric parameters (Å, °)

**Table:**

Br1—C1	1.902 (5)	C5—H5	0.9300
Br2—C4	1.894 (5)	C6—H6	0.9300
C1—C2	1.365 (6)	C7—O1	1.438 (5)
C1—C6	1.391 (7)	C7—H7A	0.9700
C2—C3	1.417 (7)	C7—H7B	0.9700
C2—C7	1.504 (6)	C8—O2	1.439 (7)
C3—C4	1.392 (7)	C8—H8A	0.9700
C3—C8	1.502 (7)	C8—H8B	0.9700
C4—C5	1.368 (8)	O1—H1	0.81 (6)
C5—C6	1.359 (9)	O2—H2	0.82 (6)
			
C2—C1—C6	122.0 (5)	C5—C6—H6	120.2
C2—C1—Br1	121.4 (4)	C1—C6—H6	120.2
C6—C1—Br1	116.5 (4)	O1—C7—C2	110.6 (4)
C1—C2—C3	118.7 (4)	O1—C7—H7A	109.5
C1—C2—C7	121.9 (4)	C2—C7—H7A	109.5
C3—C2—C7	119.4 (4)	O1—C7—H7B	109.5
C4—C3—C2	117.8 (4)	C2—C7—H7B	109.5
C4—C3—C8	122.7 (4)	H7A—C7—H7B	108.1
C2—C3—C8	119.4 (4)	O2—C8—C3	111.7 (4)
C5—C4—C3	122.3 (5)	O2—C8—H8A	109.3
C5—C4—Br2	117.0 (4)	C3—C8—H8A	109.3
C3—C4—Br2	120.7 (4)	O2—C8—H8B	109.3
C6—C5—C4	119.7 (5)	C3—C8—H8B	109.3
C6—C5—H5	120.2	H8A—C8—H8B	107.9
C4—C5—H5	120.2	C7—O1—H1	107 (5)
C5—C6—C1	119.5 (5)	C8—O2—H2	98 (5)
			
C6—C1—C2—C3	−0.1 (7)	C8—C3—C4—Br2	−1.0 (6)
Br1—C1—C2—C3	178.9 (3)	C3—C4—C5—C6	−0.1 (8)
C6—C1—C2—C7	−178.9 (4)	Br2—C4—C5—C6	179.8 (4)
Br1—C1—C2—C7	0.1 (6)	C4—C5—C6—C1	−0.6 (8)
C1—C2—C3—C4	−0.6 (6)	C2—C1—C6—C5	0.7 (8)
C7—C2—C3—C4	178.2 (4)	Br1—C1—C6—C5	−178.4 (4)
C1—C2—C3—C8	−178.8 (4)	C1—C2—C7—O1	98.1 (5)
C7—C2—C3—C8	0.0 (6)	C3—C2—C7—O1	−80.7 (5)
C2—C3—C4—C5	0.7 (7)	C4—C3—C8—O2	−114.8 (5)
C8—C3—C4—C5	178.9 (5)	C2—C3—C8—O2	63.3 (6)
C2—C3—C4—Br2	−179.2 (3)		

### Hydrogen-bond geometry (Å, °)

**Table:**

*Cg*1 is the centroid of the C1–C6 ring.

**Table:**

*D*—H···*A*	*D*—H	H···*A*	*D*···*A*	*D*—H···*A*
C8—H8A···Br2	0.97	2.61	3.192 (6)	119
C7—H7B···Br1	0.97	2.66	3.169 (5)	114
O2—H2···O1^i^	0.82 (6)	1.92 (3)	2.703 (6)	160 (7)
O1—H1···O2	0.81 (6)	1.95 (4)	2.703 (6)	152 (7)
C8—H8B···Cg1^ii^	0.97	3.01	3.63 (5)	123

Symmetry codes: (i) −*x*+1, *y*−1/2, −*z*+1/2; (ii) *x*+1, *y*, *z*.
